# Construction of an original anoikis-related prognostic model closely related to immune infiltration in gastric cancer

**DOI:** 10.3389/fgene.2022.1087201

**Published:** 2023-01-04

**Authors:** Zhihong Zhao, Cun Li, Ye Peng, Rui Liu, Qian Li

**Affiliations:** Department of Gastroenterology, Xiangya Hospital Central South University, Changsha, China

**Keywords:** gastric cancer, anoikis-related genes, prediction, prognosis, immune infiltration

## Abstract

**Background:** Anoikis is considered as a particular type of programmed cell death, the weakness or resistance of which contributes greatly to the development and progression of most malignant solid tumors. However, the latent impact of anoikis-related genes (ARGs) on gastric cancer (GC) is still ambiguous. Based on these, this study established an anoikis-related prognostic model of GC to identify the prognosis of patients and provide more effective treatment in clinical practice.

**Methods:** First, we extracted four public datasets containing the gene expression and clinicopathological information of GC, which were worked as the training and validating sets, separately. Then, an anoikis-related survival-predicted model of GC was developed *via* Lasso and COX regression analyses and verified by using the Kaplan-Meier (KM) curve and receiver operating characteristic (ROC) curve analyses. Next, we assigned GC patients to two groups characterized by the risk score calculated and analyzed somatic mutation, functional pathways, and immune infiltration between the different two groups. Finally, a unique nomogram was offered to clinicians to forecast the personal survival probability of GC patients.

**Results:** Based on seven anoikis-related markers screened and identified, a carcinogenic model of risk score was produced. Patients placed in the high-score group suffered significantly worse overall survival (OS) in four cohorts. Additionally, the model revealed a high sensitivity and specificity to prognosticate the prognoses of GC patients [area under the ROC curve (AUC) at 5-year = 0.713; GSE84437, AUC at 5-year = 0.639; GSE15459, AUC at 5-year = 0.672; GSE62254, AUC at 5-year = 0.616]. Apart from the excellent predictive performance, the model was also identified as an independent prediction factor from other clinicopathological characteristics. Combining anoikis-related prognostic model with GC clinical features, we built a more comprehensive nomogram to foresee the likelihood of survival of GC patients in a given year, showing a well-accurate prediction performance.

**Conclusion:** In summary, this study created a new anoikis-related signature for GC, which has potentially provided new critical insights into survival prediction and individualized therapy development.

## Introduction

Statistically, gastric cancer (GC) ranks fifth in morbidity and fourth in mortality in all malignant solid tumors in the whole world (Sung et al., 2021). Though the rapid advancement of endoscopic technology facilitates the early diagnosis of GC, the prognosis of patients has not significantly improved, owing to the non-specific symptoms and notorious aggressiveness of GC (Krejs, 2010; Li et al., 2022a; He et al., 2022). The vast majority of death from cancer is not due to a primary tumor but a sequel of metastatic cells within the tumor disorder (Sethi and Kang, 2011; Adeshakin et al., 2021). Consequently, identifying effectual metastasis-related prognostic biomarkers is vital to early intervention and prognosis prediction of GC.

Anoikis, a particular form of apoptosis, is stimulated by the absence of the attachment between cells or between cells and nearby extracellular matrix (Frisch and Francis, 1994; Paoli et al., 2013). It prevents dysplastic cells, pre-cancerous epithelial cells, from departing from their primary location and spreading elsewhere, avoiding the aggressive behavior of the detached tumor cells (Taddei et al., 2012). Anoikis resistance, which is the breakdown or avoidance of anoikis, is expected to confer selective superiority upon the detached cancer cells, affording them an increased anchorage-independent survival time, thereby facilitating eventual reattachment and uncontrolled growth of other sites (Frisch and Screaton, 2001; Guadamillas et al., 2011; Khan et al., 2022). Anoikis is envisioned as a pivotal defense in combating tumor metastasis and maintaining normal tissue homeostasis (Kim et al., 2012; Paoli et al., 2013). However, few studies have evaluated anoikis-related signatures in GC.

Thus, in this study, we concentrated on the predictive performance of anoikis-related genes (ARGs) in the prognosis of GC and developed an anoikis-related risk score model. We further explored and compared the differences in the genetic mutation, functional enrichment, and immune microenvironment between the two risk groups.

## Materials and methods

### Data acquisition and preprocessing

The RNA-sequencing and relevant clinical information data of GC patients used as a training set were downloaded from The Cancer Genome Atlas (TCGA) database (https://portal.gdc.cancer.gov/). All of the raw counts were transformed to transcripts per million (TPM) and log2-modified before analysis. For validation, three microarray datasets (GSE84437, GSE15459, GSE62254) along with related clinical data were acquired from Gene Expression Omnibus (GEO) database (https://www.ncbi.nlm.nih.gov/geo/) and easyGEO database (https://easygeo.cn/). The raw data of GSE84437 were quantile normalized and log2-modified before analysis. After removing duplication, 740 ARGs were integrated from the GeneCards database (https://www.genecards.org/), Harmonizome database (https://maayanlab.cloud/Harmonizome/), and National Center for Biotechnology Information (NCBI) database (https://www.ncbi.nlm.nih.gov/) (Supplementary Table S1) (Rouillard et al., 2016).

### Identification of anoikis-related prognostic markers

First, we intersected the gene symbols from TCGA-STAD and GSE84437 cohorts to guarantee that the genes achieved from the following analysis were shared and removed the batch effect between the data of two datasets by operating the “sva” R package to ensure the comparability. Then, the “limma” R package was utilized to analyze the genes with differences in expression between tumor and adjacent normal tissues in the TCGA cohort (Ritchie et al., 2015). Setting the criteria of absolute fold change (|logFC|) > 1.0 and adjusted *p*-value <0.05, we selected 1,482 differentially expressed genes (DEGs). Further, taking the intersection of ARGs and DEGs, extracting the expression matrix of intersectant genes, and combining the matched survival information, univariable Cox regression analysis was performed on TCGA-STAD and GSE84437 cohorts separately to pick out potential genes affecting the outcome of GC patients (*p* < 0.05). The Venn diagram was depicted to show the intersectant genes *via* the “VennDiagram” R package.

### Functional enrichment analysis

Based on anoikis-related DEGs, Gene Ontology (GO), Kyoto Encyclopedia of Genes and Genomes (KEGG), and Gene Set Enrichment Analysis (GSEA) enrichment analyses were conducted to seek out underlying functional pathway, by using multiple R packages (“clusterProfiler”, “enrichplot”, and “ggplot2”) (Kanehisa and Goto, 2000; Subramanian et al., 2005; Kanehisa et al., 2021). Two gene sets (“c2.cp.kegg.v2022.1.Hs.symbols.gmt”, “h.all.v2022.1.Hs.symbols.gmt”) were collected from the Molecular Signatures Database (https://www.gsea-msigdb.org/gsea/msigdb) for GSEA analysis (Subramanian et al., 2005).

### Risk score calculation

The TCGA-STAD data were worked as the training set as noted before. We made use of The Least Absolute Shrinkage and Selection Operator (LASSO) Cox regression technology to identify the promising prognostic markers and produce the anoikis-related gene prognostic score (ARGPS) model. The expression level of the candidate genes and the corresponding regression coefficients were employed as the key components of the models. The formula for calculating ARGPS is ARGPS = ∑ (regression coefficient of gene_n_ × expression level of gene_n_).

### Development and validation of the ARGPS model

Using the median ARGPS as the cut-off value, we divided 335 GC patients into high-and low-risk groups and plotted Kaplan-Meier (KM) survival curves to probe into the significant differences in the overall survival (OS) between the two groups. The prognostic value of the ARGPS model was assessed through receiver operating characteristic (ROC) curves. By computing the area under the ROC curve (AUC) in a given year in R software, we can estimate the efficiency and accuracy of the model. As for three GEO datasets (GSE84437, GSE15459, GSE62254), the validating sets, the same processes were applied to test the predictive performance of the ARGPS system. Moreover, we adopted univariable and multivariable Cox regression analyses to evaluate the independent prognosis-related significance of this model. A nomogram was made to probably calculate the survival probability for GC patients. The C-index, calibration curve, and decision curve analysis (DCA) were served to estimate the performance and credibility of the nomogram (Vickers and Elkin, 2006; Fitzgerald et al., 2015; Kerr et al., 2016; Vickers et al., 2016).

### Immune cell infiltration analysis

The CIBERSORT, a computational method, and Single-sample GSEA (ssGSEA), an extension of Gene Set Enrichment Analysis (GSEA), were applied synergistically to contrast the tumor immune microenvironment between the two groups (Newman et al., 2015). A leukocyte gene signature matrix file gained from CIBERSORTx website (https://cibersortx.stanford.edu/), was engaged to clarify the genetic signatures of 22 traditional immune cells. The four R packages (“GSVA”, “GSEABase”, “limma” and “Hmisc”) and two websites [TIMER (https://cistrome.shinyapps.io/timer/) and TIMER 2.0 (http://timer.cistrome.org/)] were exploited to measure the correlation between markers, markers and immune cells (Ritchie et al., 2015; Li et al., 2016; Li et al., 2017; Li et al., 2020).

### Mutation analysis

The somatic mutation of GC patients in the TCGA cohort was also obtained from the TCGA database (https://portal.gdc.cancer.gov/). The differences in somatic mutation data between the two risk groups were examined and took the form of waterfall graphs. The “maftool” R package was applied to calculate tumor mutation burden (TMB), referring to the number of tumor mutations per megabase in each tumor sample.

### Statistical analysis

R software version 4.2.0 served as the tool for statistical analyses. *p*-value <0.05 was viewed as statistically significant.

## Results

### Identification of anoikis-related prognostic genes

**FIGURE 1:**
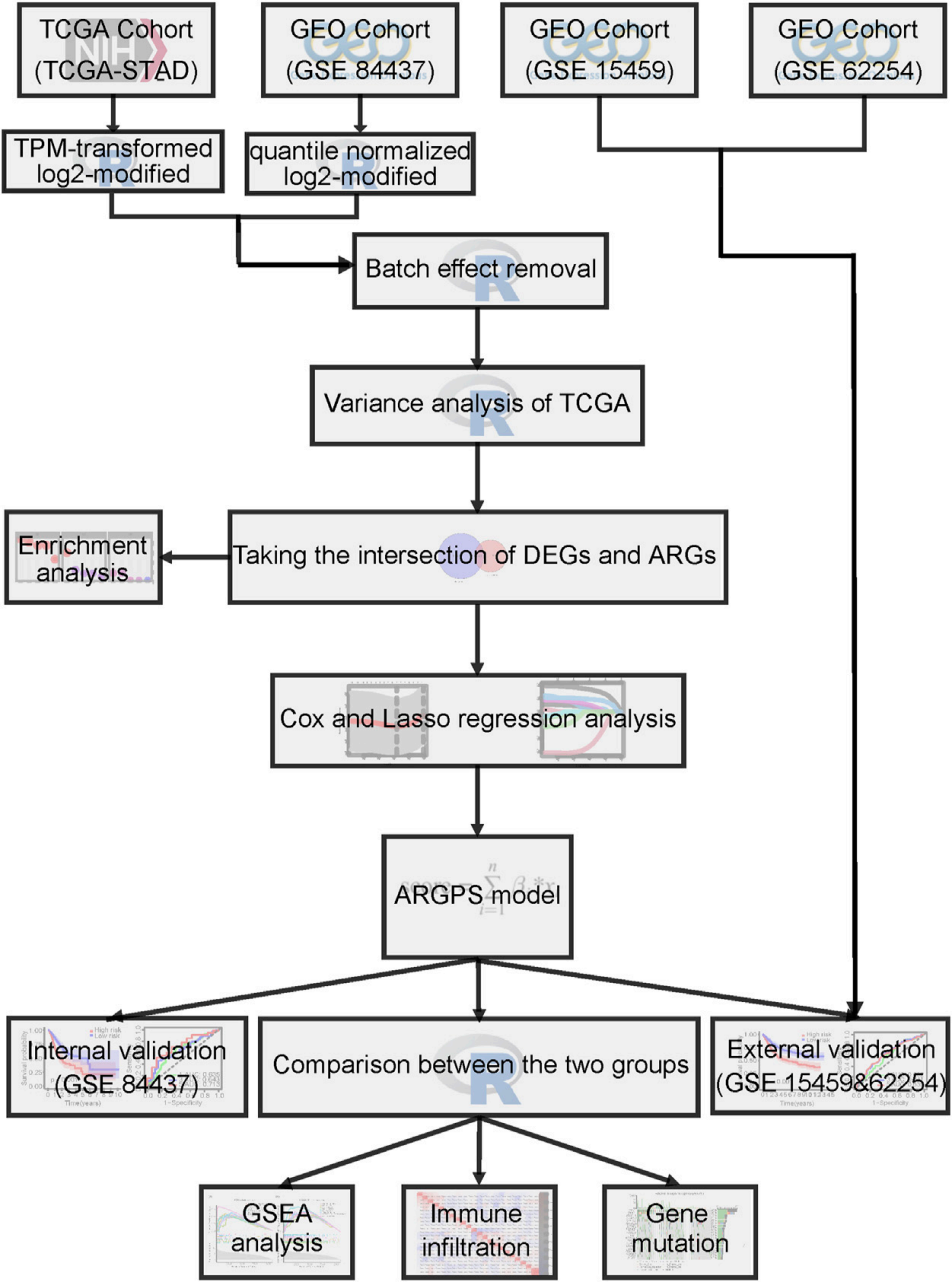
Drawing of the flow chart in this study.

**FIGURE 2:**
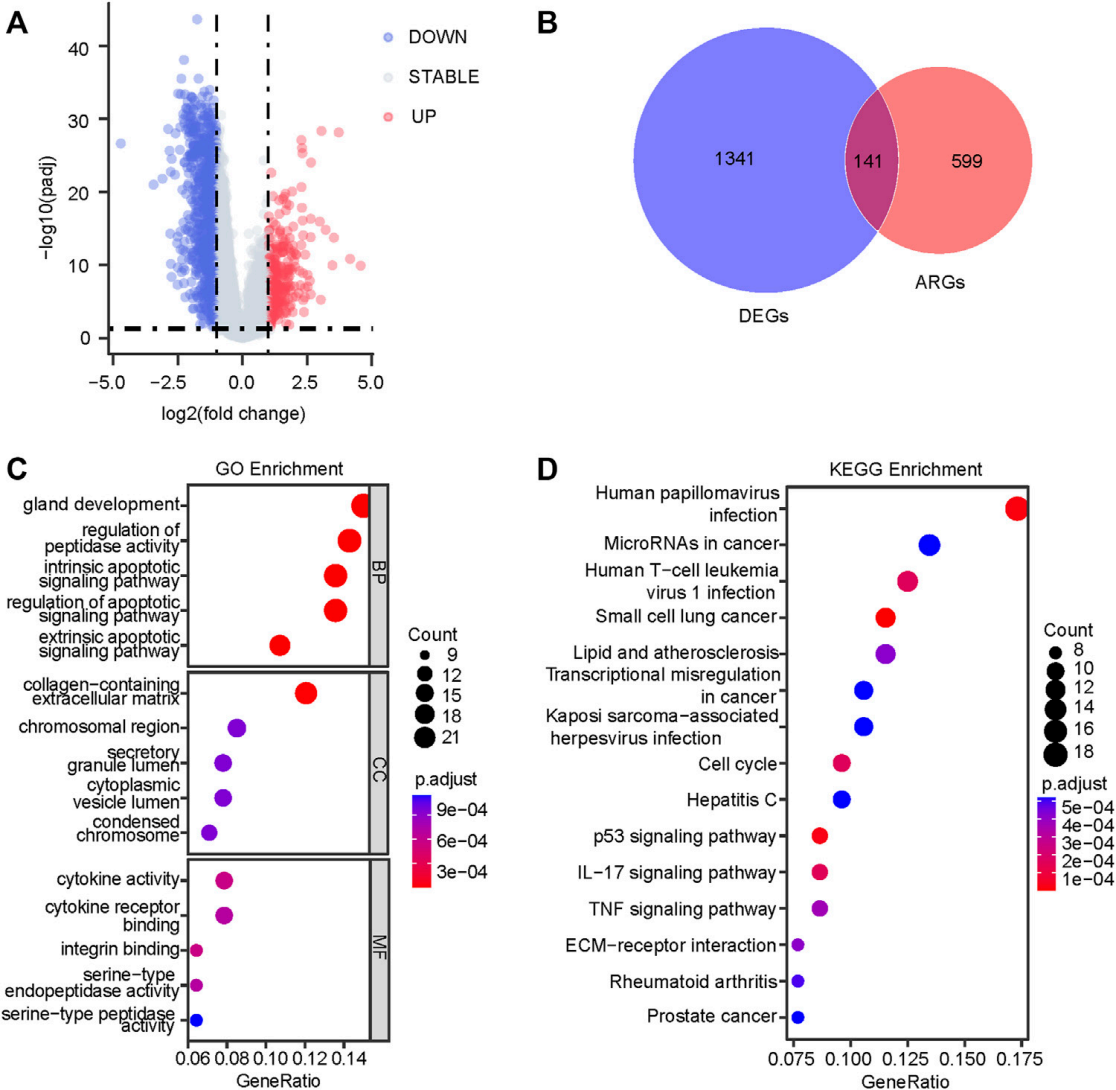
Identification of the anoikis-related prognostic genes. **(A)** The variance analysis between tumor and adjacent normal tissues in the TCGA dataset (|logFC| > 1.0, p.adj< 0.05). **(B)** The insection between 1482 DEGs and 740 ARGs is displayed by the Venn diagram. The dot plots of GO **(C)** and KEGG **(D)** enrichment analysis are based on 141 anoikis-related DEGs. TCGA, The Cancer Genome Atlas; DEGs, differentially expressed genes; ARGs, anoikis-related genes; GO, Gene Ontology; KEGG, Kyoto Encyclopedia of Genes and Genomes.

**FIGURE 3:**
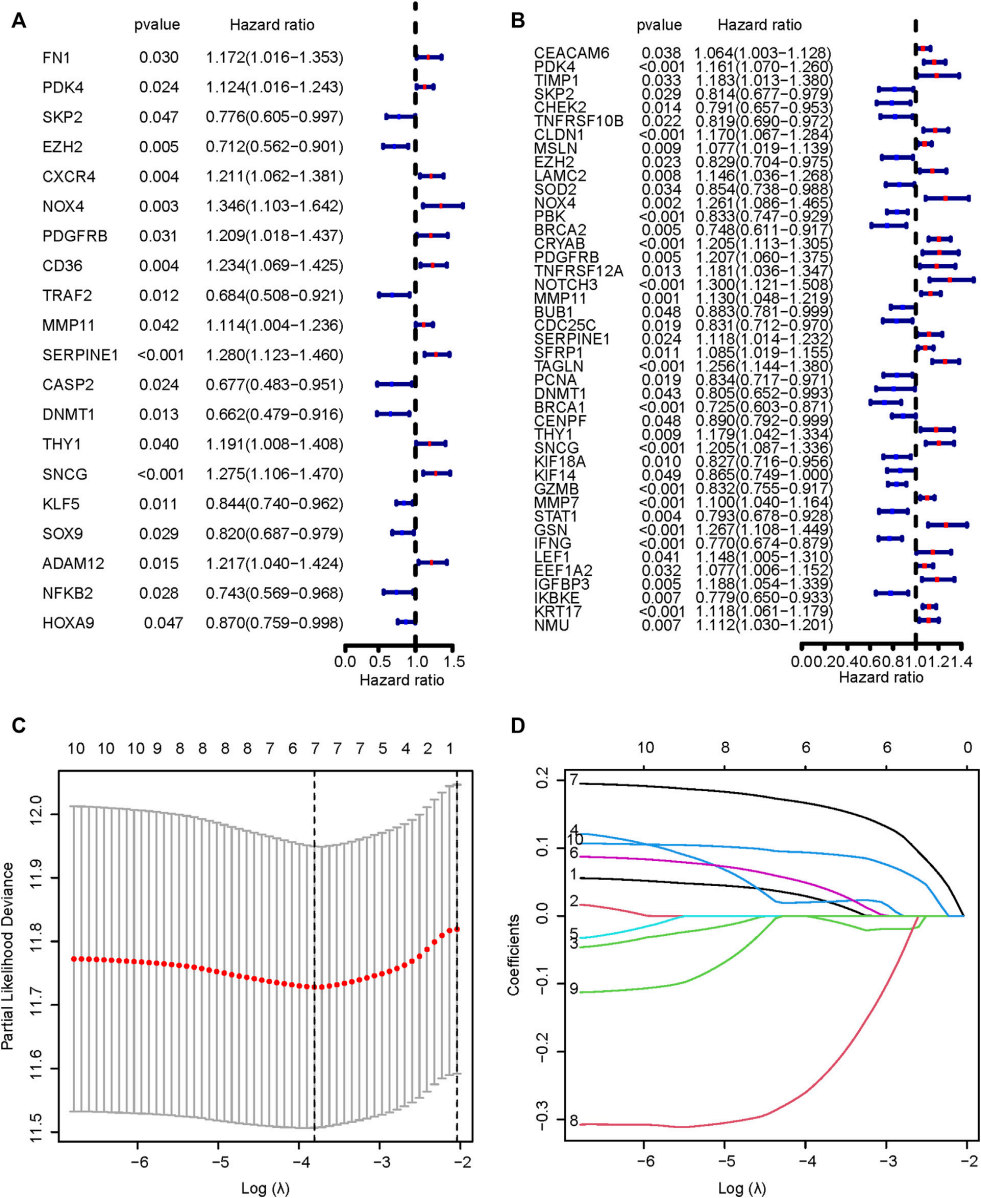
Univariable Cox regression analysis. The results of univariable Cox regression analyses of TCGA **(A)** and GEO **(B)** cohorts. **(C,D)** The results of LASSO analysis of ten prognostic ARGs. TCGA, The Cancer Genome Atlas; GEO, Gene Expression Omnibus; LASSO, Least Absolute Shrinkage and Selection Operator; ARGs, anoikis-related genes.

Figure 1 displayed the flow diagram of this study. 15,121 genes were retained through batch effect removal. 1482 DEGs were filtered in the variance analysis between cancerous and adjacent normal samples in the TCGA dataset (|logFC| > 1.0, p.adj< 0.05) (Supplementary Table S2; Figure 2A). Then, we got 141 anoikis-related DEGs by intersecting DEGs with ARGs, which was displayed by the Venn diagram (Figure 2B). GO and KEGG functional enrichment analysis on these genes were carried out to scrutinize the function of the ARGs on GC development. The result of GO analysis revealed that they were enriched in the intrinsic, extrinsic, and regulated apoptotic signaling pathways in the biological process part, and collagen-containing extracellular matrix, an indispensable substance for anoikis, in the cell component part, signifying that anoikis played a huge part in the development of GC (Figure 2C). In the KEGG analysis, the most plenteous pathways were “Human papillomavirus infection”, “MicroRNAs in cancer” and “Human T-cell leukemia virus one infection” (Figure 2D). By performing a univariable Cox regression analysis on GC patients of TCGA-STAD and GSE84437 cohorts, we gained 20 and 43 ARGs significantly associated with GC prognosis, separately. The forest plots described the detail (Figures 3A,B).

### Development and validation of the ARGPS model

**FIGURE 4:**
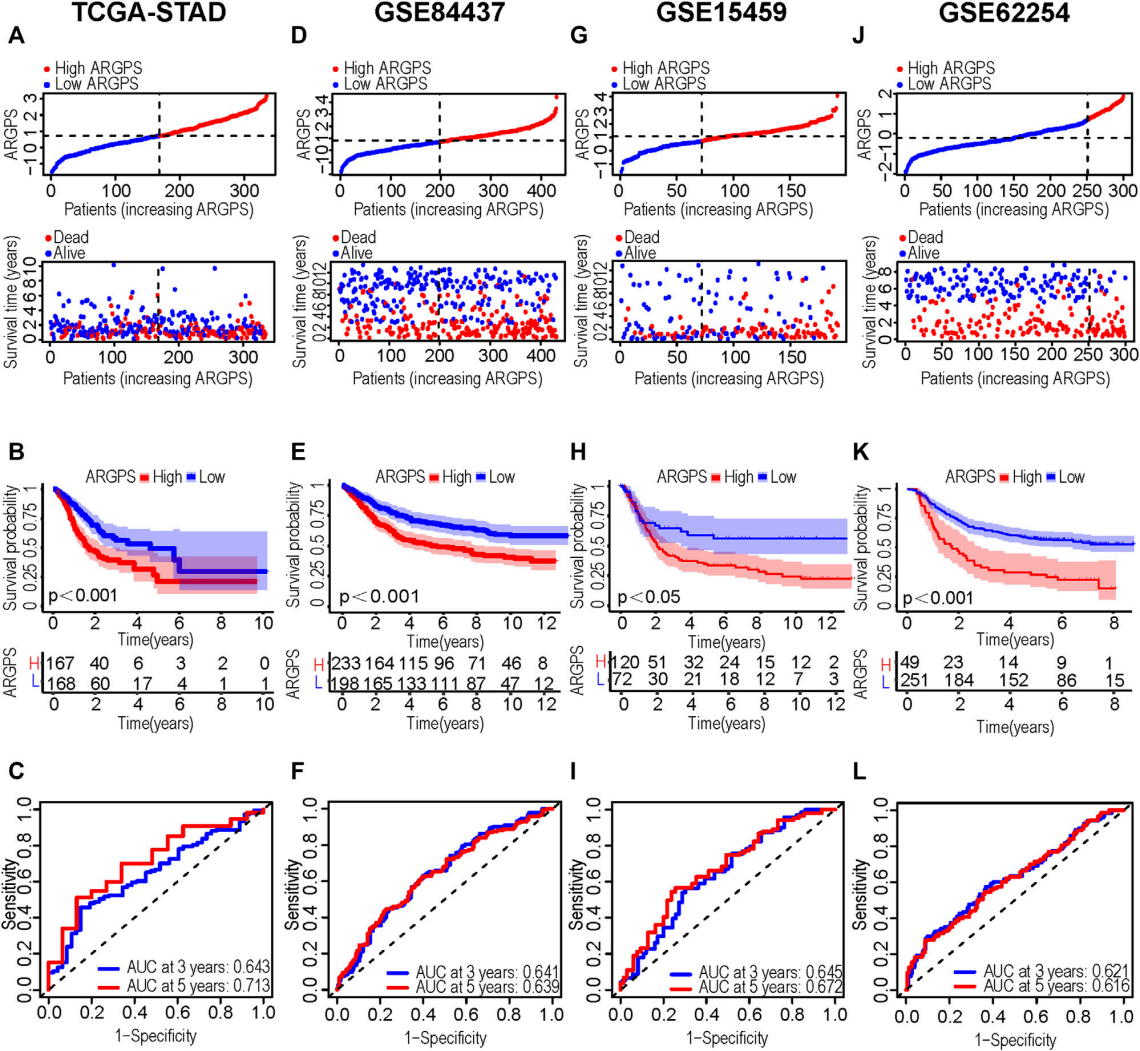
Construction and validation of the ARGPS model. **(A,D, G,J)** Distribution of ARGPS and relationship between ARGPS and survival status in the training and three testing sets. **(B,E,H,K)** The K-M survival curves of the high- and low-ARGPS groups in the training and three testing sets. **(C,F,I,L)** The time-dependent ROC curves for predicting OS at 3 and 5 years in the training and three testing sets. ARGPS, anoikis-related gene prognostic score; KM, Kaplan-Meier; ROC, receiver operating characteristic; OS, overall survival.

We intersected the results of two univariable Cox regression analyses and got 10 potential ARGs markers (PDK4, SKP2, EZH2, NOX4, PDGFRB, MMP11, SERPINE1, DNMT1, THY1, SNCG). Taking the TCGA cohort as the training set, lasso Cox regression was carried out on the ten candidate genes to identify the prognostic markers (Figures 3C,D). According to the regression analysis result, an ARGPS model was established as follows: ARGPS = 0.116 ✕ PDK4 exp + (−0.340) ✕ EZH2 exp +0.297 ✕ NOX4 exp +0.108 ✕ MMP11 exp +0.247 ✕ SERPINE1 exp + (−0.412) ✕ DNMT1 exp +0.243 ✕ SNCG exp. Based on the median ARGPS, GC patients of the TCGA cohort were classified into the high- and low-risk groups. The risk score distribution and scatter plots were mapped to indicate that GC patients with a high-risk score, had shorter survival times and a higher proportion of death (Figure 4A). Then, the KM curve illustrated that the OS of patients in the high-risk group was lower, meaning a poorer prognosis (Figure 4B). Next, we calculated the three- and five-year AUC values under the time-dependent ROC curves were 0.643 and 0.713, respectively, suggesting specificity and sensitivity of the ARGPS in prognostic prediction (Figure 4C). Furthermore, to evaluate whether the ARGPS model is suitable for other datasets, we selected GSE84437 and two additional independent GEO datasets (GSE15459, GSE62254) as validation cohorts, grouped GC patients, and did the same analyses. The same results as the training set (TCGA) were also observed, proving the excellent stability and predictive efficacy of the ARGPS (Figures 4D–L). The clinical characteristics of GC patients in four cohorts were shown in Table 1.

**TABLE 1:** Clinical characteristics of GC patients in TCGA and three GEO cohorts.

Characteristics	TCGA-STAD (*n* = 335)	GSE84437 (*n* = 433)	GSE15459 (*n* = 192)	GSE62254 (*n* = 300)
	No. of patients (%)	No. of patients (%)	No. of patients (%)	No. of patients (%)
Age				
≤65	153 (45.67)	283 (65.36)	101 (52.60)	172 (57.33)
>65	179 (53.43)	150 (34.64)	91 (47.40)	128 (42.67)
unknown	3 (00.90)	0 (0.00)	0 (0.00)	0 (0.00)
Gender				
Male	217 (64.78)	296 (68.36)	125 (65.10)	199 (66.33)
Female	118 (35.22)	137 (31.64)	67 (34.90)	101 (33.67)
Grade				
G1	9 (2.69)	NA	NA	NA
G2	120 (35.82)	NA	NA	NA
G3	197 (58.80)	NA	NA	NA
Unknown	9 (2.69)	NA	NA	NA
Stage				
Stage I	44 (13.13)	NA	31 (16.15)	30 (10.00)
Stage II	107 (31.94)	NA	29 (15.10)	96 (32.00)
Stage III	137 (40.90)	NA	72 (37.50)	95 (31.67)
Stage IV	33 (9.85)	NA	60 (31.25)	77 (25.66)
Unknown	14 (4.18)	NA	0 (0.00)	2 (0.67)
T				
T1	15 (4.48)	11 (2.54)	NA	0 (0.00)
T2	73 (21.79)	38 (8.78)	NA	186 (62.00)
T3	155 (46.27)	92 (21.24)	NA	91 (30.33)
T4	88 (26.27)	292 (67.44)	NA	21 (7.00)
Unknown	4 (1.19)	0 (0.00)	NA	2 (0.67)
N				
N0	98 (29.25)	80 (18.48)	NA	38 (12.67)
N1	91 (27.17)	188 (43.42)	NA	131 (43.67)
N2	67 (20.00)	132 (30.49)	NA	80 (26.66)
N3	68 (20.30)	33 (7.62)	NA	51 (12.00)
Unknown	11 (3.28)	0 (0.00)	NA	0 (0.00)
M				
M0	302 (90.15)	NA	NA	273 (91.00)
M1	21 (6.27)	NA	NA	27 (9.00)
Unknown	12 (3.58)	NA	NA	0 (0.00)

TCGA, The Cancer Genome Atlas; GEO, Gene Expression Omnibus.

### Validation of a nomogram

**FIGURE 5:**
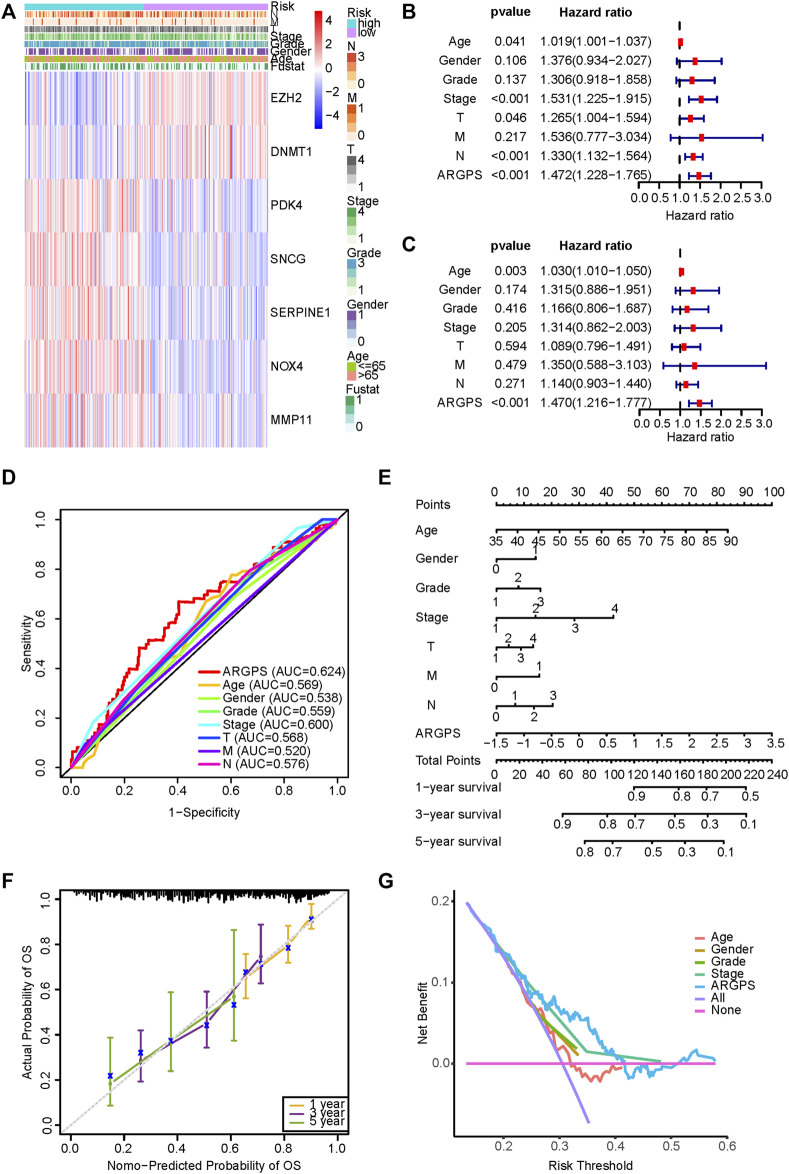
Validation of ARGPS’s ability to predict the prognosis of gastric cancer. **(A)** The differences in the expression of seven markers and the distribution of clinicopathological features between the two risk groups in the TCGA cohort were plotted by the heat map. The results of univariable **(B)** and multivariable Cox regression analysis **(C)** between ARGPS and clinicopathological factors. **(D)** The ROC curves based on the ARGPS model and other clinicopathological factors in the TCGA cohort. **(E)** Nomogram based on ARGPS and clinicopathological features in the TCGA cohort. **(F)** Calibration curves for the validation of the nomogram. **(G)** DCA curves of the clinical utility between ARGPS and other clinical factors regarding the overall survival (OS) in the TCGA cohort. TCGA, The Cancer Genome Atlas; ARGPS, anoikis-related gene prognostic score; ROC, receiver operating characteristic; DCA, Decision curve analysis.

A heat map illustrated the differences in the seven model genes expression and the distribution of clinicopathological features between two risk groups in the training set (Figure 5A). In combination with the clinical features of GC patients, we performed the univariable and multivariable Cox regression analyses and the result showed the independent prognostic predictability of the ARGPS (Figures 5B–D). Given the inconvenient clinical utility of the ARGPS, a hybrid nomogram model was created for predicting the survival probability of GC patients in a given year (Figure 5E). The result showed that C-index was 0.687, denoting the great reliability of the nomogram. Calibration curves of the OS at 1, 3, and 5 years were evenly distributed diagonally, proving the pretty fitness of the model (Figure 5F). Additionally, from DCA curves and AUC values, in clinical decision-making, the ARGPS model was found to be able to serve as the most effective prognostic indicator among other clinical characteristics (Figures 5D,G).

### ARGPS model and functional analysis, gene mutation

**FIGURE 6:**
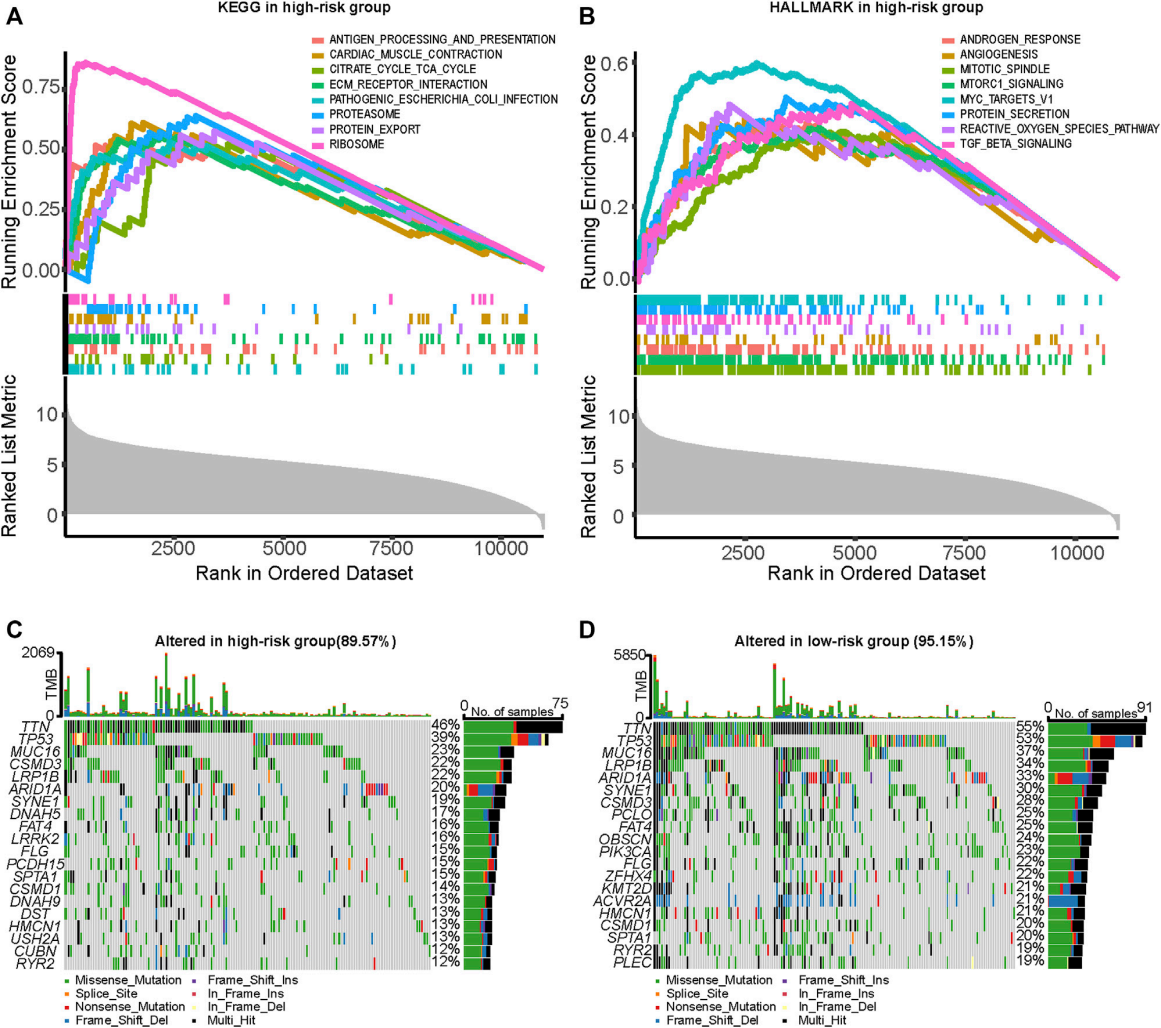
Analysis of enrichment function and genetic mutation at a different risk score. **(A,B)** Part of significantly upregulated pathways in the high-risk group enriched by GSEA analysis. **(C,D)** Comparison of genetic mutation between the high- and low-risk groups utilized by the “maftool” R package. GSEA, Gene Set Enrichment Analysis.

For the purpose of further elucidating the underlying mechanisms of the impact of ARGPS on prognosis, KEGG and HALLMARK gene sets were selected to search for significantly enriched pathways between the two risk groups. In the high-risk group, the genes were mostly enriched in antigen processing and presentation, extracellular matrix (ECM) receptor interaction, protein export, proteasome, and ribosome in the KEGG part, and angiogenesis, mitotic spindle, protein secretion, reactive oxygen species pathway, and TGF-beta signaling in the HALLMARK part (Figures 6A,B). Detailed enrichment pathways and parameters are shown in Supplementary Tables S3, S4. Waterfall plots were exploited to analyze the somatic mutations in the two risk groups. From Figures 6C,D, the most common type of mutations in both groups was missense mutations, followed by Multi_Hit, which means that a gene has multiple mutations in the same sample. In the high-risk group, the overall levels of TMB were lower than those in the other group, which is contrary to our conventional understanding. Besides that, all mutant genes shown in the graphs were mutated less frequently in the high-risk group.

### ARGPS model and immune infiltration

**FIGURE 7:**
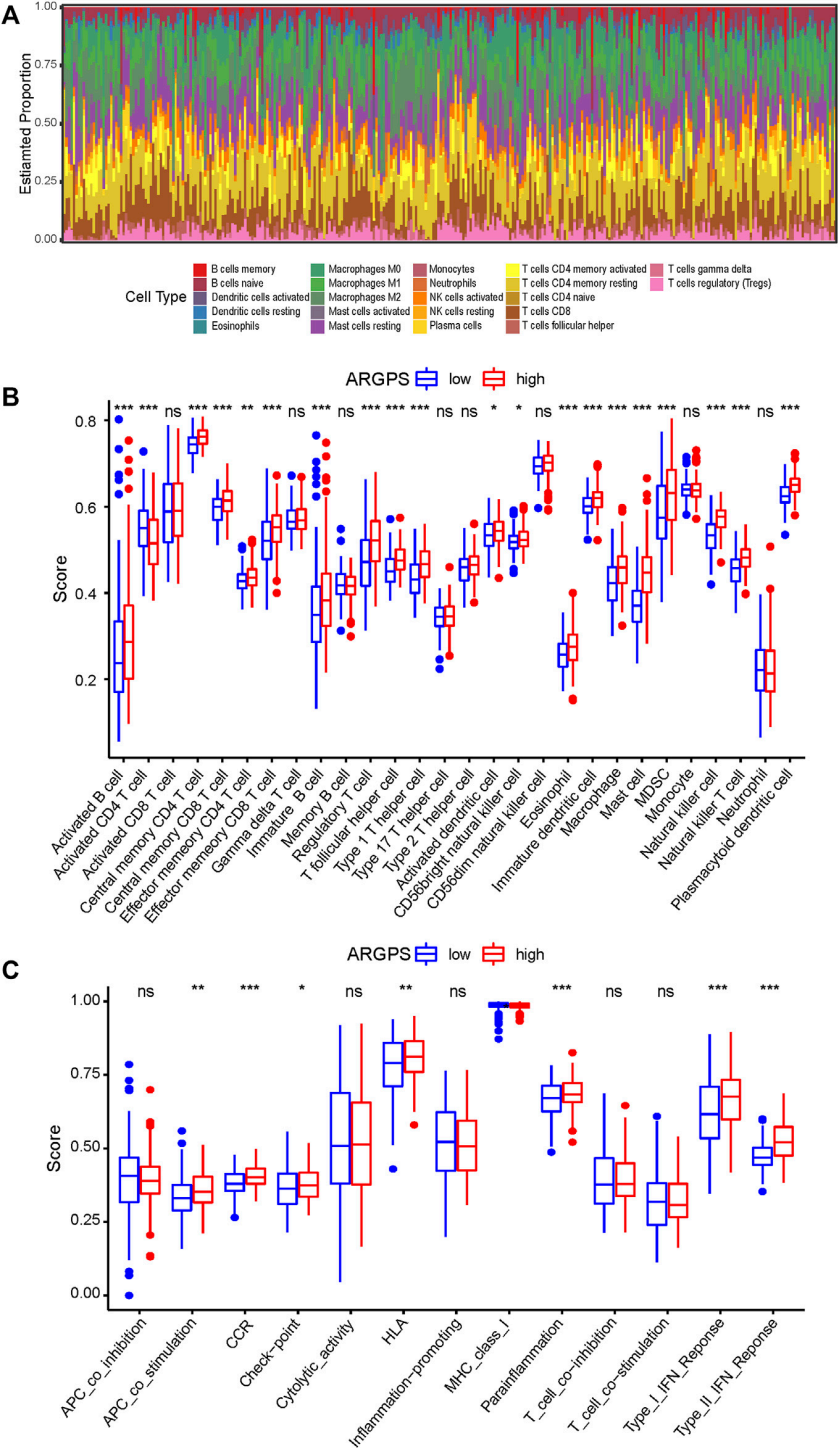
Evaluation of the immune microenvironment of gastric cancer. **(A)** The proportion and distribution of 22 immune cells in each sample of the TCGA cohort were calculated by the CIBERSORT algorithm. The sum of all estimated cell scores in each sample is 1. The difference of **(B)** 28 immune cells and **(C)** 13 immune pathways infiltration levels between the high- and low-risk groups compared by the ssGSEA analysis. ns > 0.05, *<0.05, **<0.01, ***<0.001. TCGA, The Cancer Genome Atlas; ssGSEA, Single-sample Gene Set Enrichment Analysis.

**FIGURE 8:**
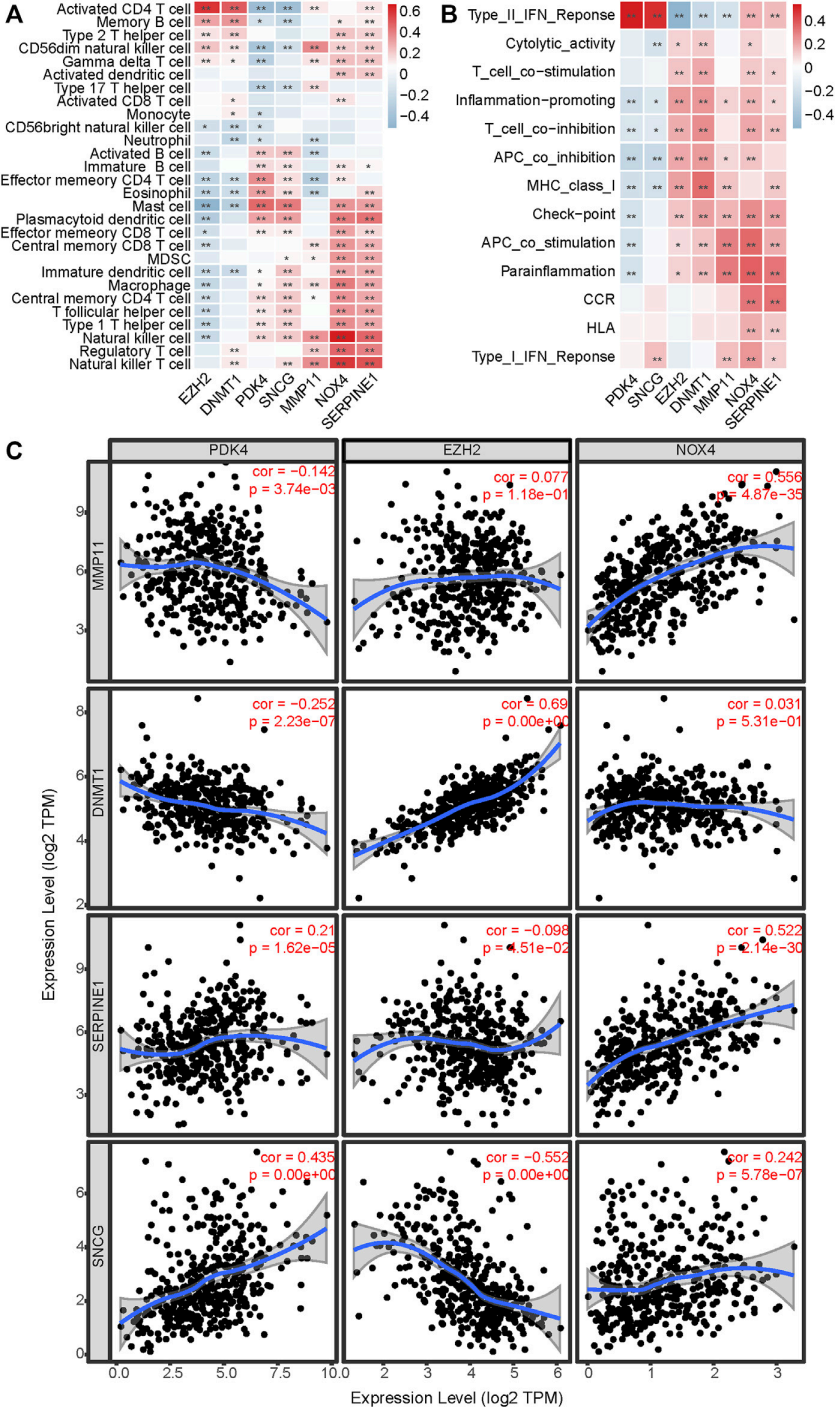
Estimation of the correlation between markers and immune infiltration. The correlation between the seven markers and **(A)** 28 immune cells and **(B)** 13 immune pathways showed by heat maps. **(C)** The linear relation of each of the two markers in the model is predicted by TIMER. *< 0.05, **< 0.01.

To explore whether and how the ARGPS model influenced the tumor immune landscape, bar graphs were first drawn to show the relative proportion of 22 different immune cells in every sample of the TCGA cohort (Figure 7A). The ssGSEA analysis was applied to study deeply the discrepancy between the immune status of the two risk groups. For type analysis of 28 immune cells, we discovered that compared to GC patients with a lower risk score, those with a higher risk score had significantly higher infiltration of multiple cells (including activated B cell, central and effector memory T cell, immature B cell, regulatory T cell, T follicular helper cell, type 1 T helper cell, activated dendritic cell, CD56 bright natural killer cell, eosinophil, immature dendritic cell, macrophage, mast cell, MDSC, natural killer cell, natural killer T cell, and plasmacytoid dendritic cell), whereas lower infiltration of activated CD4 T cell (Figure 7B). For type analysis of 13 immune pathways, multiple pathways (including APC co-stimulation, CCR, check-point, HLA, parainflammation, type I and II interferon response) of the high-risk group were also significantly more vibrant than those of the low-risk group, which may work for the worse prognosis of the GC patients (Figure 7C). Furthermore, the heat maps were painted to show the strong relationship of the seven markers to immune cells and pathways (Figures 8A,B). In addition, the TIMER database was available to predict the relation between the markers. Figure 8C plotted the linear correlation of each of the two markers, indicating the intense relationship between the seven markers.

## Discussion

Previously, there were some reports in the literature about the effects of anoikis on GC. Kai Wang et al. sequenced the whole genomes of gastric cancer tissues and performed comprehensive molecular profiling, discovering that RHOA hotspot mutants could facilitate anoikis escape in the organoid cultures (Wang et al., 2014a). Numbers of molecules and pathways have been confirmed to be involved in the anoikis resistance, which resulted in the metastasis and progression of GC (Li et al., 2020b; Ye et al., 2020; Zhang et al., 2022a; Li et al., 2022b). In addition, by reducing anoikis resistance and cancer cell mobility, some drugs could trigger apoptosis and inhibit metastasis, thereby delaying the progression of GC (Kim et al., 2022). All of the above emphasized that the notion of targeting genes associated with anoikis might be imperative to control tumor development and progression. As we know, in GC, this study is the first to identify anoikis-related prognostic biomarkers and construct a relevant predictive model to evaluate patient outcomes.

In this research, we screened out seven genes related to the prognosis of GC, which contained PDK4, EZH2, NOX4, MMP11, SERPINE1, DNMT1, and SNCG, and created a predictive risk score model, namely ARGPS model. Certain interactions between these markers and tumor initiation and progression have been described in studies before. For example, Zimu Zhang et al. disclosed that PDK4 promoted invasion and migration ability of GC cells (Zhang et al., 2022b). In the high-PDK4 group, enriched functional pathways were correlated with cell adhesion regulation and synaptic activity, which were substantial in cancer anoikis resistance, proliferation, invasion, and metastasis (Zhong and Rescorla, 2012; Alanko et al., 2015; Zhang et al., 2022b). *In vitro* studies demonstrated that EZH2 bound to the vital tumor suppressor PTEN locus and led to proliferation, invasion, and pluripotent phenotype of GC cells (Gan et al., 2018). IL-6/STAT3 signaling, whose aberrant expression in GC cells was thought to be a main mechanism for tumorigenesis and pathogenesis, drove EZH2 transcriptional stimulation and mediated unfortunate outcome (Yu and Jove, 2004; Yu et al., 2009; Li et al., 2010; Pan et al., 2016). NOX4, one of the major origins of reactive oxygen species (ROS), played a crucial role in genomic instability, resistance to anoikis, migration, and extravasation into distant sites (Bedard and Krause, 2007; Liou and Storz, 2010; Peiris-Pages et al., 2015; Schumacker, 2015). The expression of NOX4 in GC was significantly relevant to tumor size, lymph node metastasis, venous invasion, and unfortunate survival (Du et al., 2019). What is interesting is that NOX4 could enhance cell propagation by activating the GLI1 transcription factor, which was a distinguished molecule in the Hedgehog signaling pathway (Briscoe and Therond, 2013; Tang et al., 2018). Meanwhile, it was verified that the suppression of GLI1 protein could evoke anoikis *in vitro* and prevent tumor formation *in vivo* (Kandala and Srivastava, 2012). Similarly, SERPINE1, a key inhibitor of tissue plasminogen activator and urokinase, is abundant in tumor tissues and strongly interrelated with the propagation and invasiveness of GC cells (Chen et al., 2022). SERPINE1 could induce angiogenesis and tumor inflammatory microenvironment, in which anoikis was a critical player, by regulating the expression level of VEGF and IL-6 *via* VEGF and JAK-STAT3 inflammatory pathways (Sakamoto and Kyprianou, 2010; Feng et al., 2014; Teng et al., 2021; Chen et al., 2022). Y-B Kou et al. discovered that the growth, expansion, and invasion activities of GC cells could be inhibited by the knockdown of MMP11, probably through downregulation of the PCNA, IGF-1, and VEGF (Kou et al., 2013). In addition, MMP11 in exosomes secreted from gastric cancer-associated fibroblasts can be delivered into GC cells to partially accelerate their progression and metastasis (Xu et al., 2019). DNMT1, whose full name is DNA methyltransferase 1, is one of the DNA-modifying enzymes (Lyko, 2018). It might participate in the modulation of DNA methylation levels and give rise to the development of an anoikis-resistance phenotype (Campos et al., 2007; Lyko, 2018). Recently, a study suggested that lncRNA SAMD12-AS1 potentially played oncogenic roles in GC by directly bounding to DNMT1 and enabling DNMT1 to restrain the P53 signal pathway (Lu et al., 2021a). A strong interaction between the expression level of SNCG, a pro-metastatic oncogene, in primary and metastatic sites has been revealed in many solid tumor types (Liu et al., 2005). In addition, SNCG expression in GC tissues, particularly in metastatic tissues, was relevant to tumor microenvironment and metastasis (Hu et al., 2009; Wang et al., 2014b). Thus, hypoxia-inducible lncRNA-AK058003 could increase GC metastasis by targeting SNCG (Wang et al., 2014b). It is worth noting that markers did not work alone but had some linkages. For instance, the synergistic mediation of methylation by EZH2 and DNMT1 contributed to the progression of GC (Ning et al., 2015).

Based on ARGPS we calculated, GC patients were separated into high- and low-risk groups. Follow-up analyses revealed that the GC patients with the high-risk score correlated with a poorer prognosis, which was confirmed by three testing cohorts (GSE84437, GSE15459, GSE62254). The results of univariable and multivariable Cox analyses with other clinical confounding factors showed extraordinary standalone prediction value of ARGPS. Then, a nomogram was built to accurately quantify personalized predictive scores and survival probabilities. Both C-index and the calibration curves showed superb consistency. Additionally, decision curve analysis was used to suggest the potential clinical utility of the model.

We compared the differences in functional pathways and somatic mutations between the two groups. GSEA analyses have enriched ECM receptor interaction and reactive oxygen species pathways, which were highly related to anoikis (Tang et al., 2018). Moreover, these results suggested that anoikis might closely connect with immune invasion, material transportation, and angiogenesis in GC. Intriguingly, not only the overall pattern of gene mutations was lower in the high score group but also the mutation frequency of commonly mutated genes was lower. The difference in TMB between the two groups was confirmed to be statistically significant by the Wilcoxon rank sum test. Although most genetic mutations (such as missense mutations) were harmful or lethal to the body, the possibility of beneficial effects could not be entirely ruled out. A panel-based sequencing study of advanced gastric cancer showed that patients with elevated TMB had higher objective response rates and longer progression-free survival, suggesting that TMB could be employed as a potential predictive biomarker (Kim et al., 2020). Among patients with advanced gastric cancer who received neoadjuvant chemotherapy before radical gastrectomy, those with high TMB showed favorable treatment response and better disease-free survival (Li et al., 2021). Besides, in multiple cancer types, TMB was considered as another indicator of patients’ response to immunotherapy because a positive correlation between TMB and benefit of immunotherapy was observed in a comprehensive analysis (Hodges et al., 2017; Yarchoan et al., 2017).

Epigenomic alterations in cancer interact with the immune microenvironment to dictate tumor evolution and therapeutic response (Sundar et al., 2022). Though a variety of programmed cell death modes (e.g., necroptosis, pyroptosis, ferroptosis, *etc.*) have been showed to be associated with tumor immunity, the correlation between anoikis and immunity is still unclear (Gao et al., 2022; Niu et al., 2022). We managed to explore the differences in the immune landscape between the two groups, showing that in the high score group with the worrisome outcomes, the proportion of most immune cells and functions were significantly increased, representing that anoikis may regulate tumor progression by affecting immune infiltration levels. If we think about this among all the different immune cells, there are both protumorigenic and antitumorigenic cells. One should note that one of the most crucial elements of the tumor immunosuppressive microenvironment are myeloid-derived suppressor cells (MDSCs), which plays an important role in *Helicobacter* pylori-induced intestinal metaplasia and tumor progression (Ding et al., 2016; Ding et al., 2020; Ding et al., 2022). Based on our results, MDSCs infiltration level was relatively high in the high-ARGPS group and was significantly related to SNCG, MMP11, NOX4 and SERPINE1. Besides, EZH2 and DNMT1 could regulate the differentiation and accumulation of MDSCs (Huang et al., 2019; Smith et al., 2020; Lu et al., 2021b; Yang et al., 2022). Due to the certainty of immunity on tumor progression and the uncertainty of anoikis on the immune landscape, the interaction between anoikis and immunity (especially MDSCs) might be an interesting field to research.

Though this study has made a breakthrough, it still is limited by some aspects. First, this study was confined to mining and analyzing public databases. Second, although the established model and nomogram had a pretty good predictive capability, taking the heterogeneity of the cells in tumor tissues into consideration, studies on anoikis executed at the single-cell level may shed light on the critical role of anoikis on the outcome of GC patients more accurately. Third, despite this study showing that there was a powerful relationship between the ARGs and immunity, the detailed mechanism was still not fully explained. Finally, the study has a lack of validation *in vivo* or *in vitro*. Through combined the results of this study with previous literature, we reasonably believe that the underlying mechanism of anoikis-related markers and gastric cancer immune microenvironment (especially MDSCs) seems to be full of promises and worthful for future investigation.

## Conclusion

To sum up, our seven-gene ARGPS model is capable of predicting the outcome of GC patients, and the nomogram can assist the clinician to develop personalized treatment plans for various patients. More research in the future into the molecular interaction between anoikis and tumor is required to provide the theoretical basis for clinical practice and a road map for precision medicine.

## Data Availability

Publicly available datasets were analyzed in this study. This data can be found here: TCGA: https://portal.gdc.cancer.gov/GEO: https://www.ncbi.nlm.nih.gov/geo/.

## Glossary

APC co_stimulation: antigen-presenting cell co-inhibition
APC co_inhibition: antigen-presenting cell co-inhibition
ARGPS: anoikis-related gene prognostic score
ARGs: anoikis-related genes
AUC: area under the ROC curve
CCR: C-C chemokine receptor
DEGs: differentially expressed genes
DNMT1: DNA methyltransferase 1
EZH2: enhancer of zeste homologue 2
EMT: epithelial–mesenchymal transition
exp: expression
GC: gastric cancer
GEO: Gene Expression Omnibus
GSEA: Gene Set Enrichment Analysis
GLI1: GLI family zinc finger 1
GO: Gene Ontology
HLA: human lymphocyte antigen
IL-6: interleukin-6
IGF-1: insulin-like growth factors-1
lncRNA: long non-coding RNA
JAK: Janus tyrosine Kinase
KM: Kaplan-Meier
LASSO: least absolute shrinkage and selection operator
MMP11: matrix metalloproteinase 11
NOX4: NADPH oxidase 4
MDSC: Myeloid-derived suppressor cells
MHC_class_I: major histocompatibility complex class I
NCBI: National Center for Biotechnology Information
OS: overall survival
PCNA: proliferating cell nuclear antigen
PDK4: pyruvate dehydrogenase kinase-4
ROC: receiver operating characteristic
ROS: reactive oxygen species
STAD: stomach adenocarcinoma
TCGA: The Cancer Genome Atlas
ssGSEA: Single-sample Gene Set Enrichment Analysis
TMB: tumor mutation burden
SKP2: s-phase kinase associated protein 2
PDGFRB: platelet derived growth factor receptor Beta
SERPINE1: serine protease inhibitor clade E member 1
SNCG: γ-synuclein
STAT3: signal transducer and activator of transcription 6
THY1: Thy-1 cell surface antigen
Type_I_IFN_Reponse: type I interferon response
Type_II_IFN_Reponse: type II interferon response
VEGF: vascular endothelial growth factor.
